# A Novel Way of Feeding Pups

**Published:** 1882-07

**Authors:** George M. Parkinson


### XI.—A NOVEL WAY OF FEEDING PUPS.

REPORTED BY GEORGE M. PARKINSON, D.V.S.

My red Irish setter bitch has six pups, aged nine weeks, which she has weaned. She goes with me to my boarding place, takes her rations, and returns to her pups, vomits and lets the pups eat. The first time the act was performed, I supposed she was sick, but she constantly repeated the act.

One evening I took her to tea with me. She ate her supper, and I remained a little longer than usual, when she commenced to cry and whimper, as she always does when she wants anything or to go anywhere.

I could not imagine what was wanted, but soon found out when I got back to where her puppies were, for she had discharged the contents of her stomach, and the puppies were enjoying their supper. This may not be uncommon, but I have never witnessed it before or seen any one who has.

MIDDLETOWN, CONN., May, 1882.

